# Supplementary material for: Epidemiological Study of Adenoid Cystic Carcinoma and Its Outcomes: Insights from the Surveillance, Epidemiology, and End Results (SEER) Database
Source: Cancers (Basel). 2024 Oct 3;16(19):3383. doi: 10.3390/cancers16193383 (PMC11476411; doi:10.3390/cancers16193383)
Supplement: Supplementary file 1 [file cancers-16-03383-s001.zip › cancers-3159058-supplementary.pdf]

Legend

Supplementary Table 1: U.S. Census Bureau-designated regions and divisions

Supplementary Table 2: Details of organs/sites classification

Supplementary Table 3: Reasons for no cancer directed surgery

Supplementary Table 4: The treatment modalities among different included sites

Supplementary Table 5: Assessment of Ethnicity/Hispanic disparity among different regions

Supplementary figure 1: Distribution of race by geographic areas (percent was calculated per the entire cohort)

Supplementary figure 2: Cancer-specific survival among different organs/sites

Supplementary figure 3: Overall survival among different ethnicities.

Supplementary figure 4: Forest plot showing late mortality predictors among surgery subsets.

Supplementary Table 1: U.S. Census Bureau-designated regions and divisions (30).

Region 1: Northeast
Division 1: New England (Connecticut, Maine, Massachusetts, New Hampshire, Rhode Island, and Vermont)
Division 2: Mid-Atlantic (New Jersey, New York, and Pennsylvania)
Region 2: Midwest
Division 3: East North Central (Illinois, Indiana, Michigan, Ohio, and Wisconsin)
Division 4: West North Central (Iowa, Kansas, Minnesota, Missouri, Nebraska, North Dakota, and South Dakota)
Region 3: South
Division 5: South Atlantic (Delaware; Florida; Georgia; Maryland; North Carolina; South Carolina; Virginia; Washington, D.C. and West Virginia)
Division 6: East South Central (Alabama, Kentucky, Mississippi, and Tennessee)
Division 7: West South Central (Arkansas, Louisiana, Oklahoma, and Texas)
Region 4: West
Division 8: Mountain (Arizona, Colorado, Idaho, Montana, Nevada, New Mexico, Utah, and Wyoming)
Division 9: Pacific (Alaska, California, Hawaii, Oregon, and Washington)

Supplementary Table 2: Details of organs/sites classification

	Overall	A-Head/Neck	B-Thoracic	C-Breast	D-Genitourinary	E-Miscellaneous
n	5150	3612	348	728	114	348
Site recode ICD-O-3 WHO (%)						
Anus, Anal Canal and Anorectum	4 (0.1)	0 (0.0)	0 (0.0)	0 (0.0)	0 (0.0)	4 (1.1)
Breast	728 (14.1)	0 (0.0)	0 (0.0)	728 (100.0)	0 (0.0)	0 (0.0)
Cervix Uteri	31 (0.6)	0 (0.0)	0 (0.0)	0 (0.0)	31 (27.2)	0 (0.0)
Corpus Uteri	2 (0.0)	0 (0.0)	0 (0.0)	0 (0.0)	2 (1.8)	0 (0.0)
Esophagus	4 (0.1)	0 (0.0)	0 (0.0)	0 (0.0)	0 (0.0)	4 (1.1)
Eye and Orbit	120 (2.3)	120 (3.3)	0 (0.0)	0 (0.0)	0 (0.0)	0 (0.0)
Floor of Mouth	81 (1.6)	81 (2.2)	0 (0.0)	0 (0.0)	0 (0.0)	0 (0.0)
Gum and Other Mouth	644 (12.5)	644 (17.8)	0 (0.0)	0 (0.0)	0 (0.0)	0 (0.0)
Hypopharynx	6 (0.1)	6 (0.2)	0 (0.0)	0 (0.0)	0 (0.0)	0 (0.0)
Larynx	65 (1.3)	65 (1.8)	0 (0.0)	0 (0.0)	0 (0.0)	0 (0.0)
Lip	59 (1.1)	59 (1.6)	0 (0.0)	0 (0.0)	0 (0.0)	0 (0.0)
Lung and Bronchus	189 (3.7)	0 (0.0)	189 (54.3)	0 (0.0)	0 (0.0)	0 (0.0)
Miscellaneous	54 (1.0)	0 (0.0)	0 (0.0)	0 (0.0)	0 (0.0)	54 (15.5)
Nasopharynx	118 (2.3)	118 (3.3)	0 (0.0)	0 (0.0)	0 (0.0)	0 (0.0)
Nose, Nasal Cavity and Middle Ear	525 (10.2)	525 (14.5)	0 (0.0)	0 (0.0)	0 (0.0)	0 (0.0)
Oropharynx	11 (0.2)	11 (0.3)	0 (0.0)	0 (0.0)	0 (0.0)	0 (0.0)
Other Endocrine including Thymus	2 (0.0)	0 (0.0)	2 (0.6)	0 (0.0)	0 (0.0)	0 (0.0)
Other Female Genital Organs	1 (0.0)	0 (0.0)	0 (0.0)	0 (0.0)	1 (0.9)	0 (0.0)
Other Male Genital Organs	2 (0.0)	0 (0.0)	0 (0.0)	0 (0.0)	2 (1.8)	0 (0.0)
Other Non-Epithelial Skin	284 (5.5)	0 (0.0)	0 (0.0)	0 (0.0)	0 (0.0)	284 (81.6)
Other Oral Cavity and Pharynx	4 (0.1)	4 (0.1)	0 (0.0)	0 (0.0)	0 (0.0)	0 (0.0)
Other Urinary Organs	3 (0.1)	0 (0.0)	0 (0.0)	0 (0.0)	3 (2.6)	0 (0.0)
Ovary	1 (0.0)	0 (0.0)	0 (0.0)	0 (0.0)	1 (0.9)	0 (0.0)
Pancreas	1 (0.0)	0 (0.0)	0 (0.0)	0 (0.0)	0 (0.0)	1 (0.3)
Prostate	7 (0.1)	0 (0.0)	0 (0.0)	0 (0.0)	7 (6.1)	0 (0.0)
Salivary Gland	1735 (33.7)	1735 (48.0)	0 (0.0)	0 (0.0)	0 (0.0)	0 (0.0)
Soft Tissue including Heart	18 (0.3)	0 (0.0)	18 (5.2)	0 (0.0)	0 (0.0)	0 (0.0)
Stomach	1 (0.0)	0 (0.0)	0 (0.0)	0 (0.0)	0 (0.0)	1 (0.3)
Tongue	223 (4.3)	223 (6.2)	0 (0.0)	0 (0.0)	0 (0.0)	0 (0.0)
Tonsil	21 (0.4)	21 (0.6)	0 (0.0)	0 (0.0)	0 (0.0)	0 (0.0)
Trachea, Mediastinum and Other Respiratory Organs	139 (2.7)	0 (0.0)	139 (39.9)	0 (0.0)	0 (0.0)	0 (0.0)
Vagina	6 (0.1)	0 (0.0)	0 (0.0)	0 (0.0)	6 (5.3)	0 (0.0)
Vulva	61 (1.2)	0 (0.0)	0 (0.0)	0 (0.0)	61 (53.5)	0 (0.0)

ICD-O-3: International Classification of Diseases for Oncology, 3rd Edition, WHO: World Health Organization

Supplementary Table 3: Reasons for no cancer directed surgery

Reason	Patients
**Not recommended**	616
**Recommended but not performed, unknown reason**	56
**Recommended but not performed, patient refused**	47
**Not recommended, contraindicated due to other condition**	35
**Unknown, death certificate or autopsy**	24
**Recommended, unknown if performed**	16
**Not performed, patient died prior to recommended surgery**	1

Supplementary Table 4: The treatment modalities among different included sites

	B-Thoracic	A-Head/Neck	C-Breast	D-Genitourinary	E-Miscellaneous	p	SMD
**n**	348	3612	728	114	348		
**Surgery (%)**	236 (67.8)	3061 (84.7)	713 (97.9)	93 (81.6)	252 (72.4)	<0.001	0.412
**Radiotherapy (%)**	134 (38.5)	2230 (61.7)	372 (51.1)	37 (32.5)	72 (20.7)	<0.001	0.433
**Chemotherapy (%)**	67 (19.3)	428 (11.8)	87 (12.0)	24 (21.1)	15 (4.3)	<0.001	0.251

SMD= standardized mean difference

Supplementary Table 5: Assessment of Ethnicity/Hispanic disparity among different regions

	level	Overall	Northeast	Midwest	South	West	p	SMD
n		5150	887	217	1158	2888		
Ethnicity (%)	Non-Hispanic	4528 (87.9)	796 (89.7)	214 (98.6)	1121 (96.8)	2397 (83.0)	<0.001	0.337
	Hispanic	622 (12.1)	91 (10.3)	3 (1.4)	37 (3.2)	491 (17.0)		
	Hazard ratio (95%CI), P-value ¶	0.89 (0.77-1.04), P=0.141	0.78 (0.51-1.21), P=0.263	1.91 (0.26-13.88), P=0.521	0.68 (0.34-1.37), P=0.284	0.93 (0.79-1.10), P=0.416		
Ethnicity details (%)	Non-Hispanic American Indian/Alaska Native	29 (0.6)	1 (0.1)	0 (0.0)	2 (0.2)	26 (0.9)	<0.001	0.78
	Non-Hispanic Asian or Pacific Islander	572 (11.1)	45 (5.1)	0 (0.0)	23 (2.0)	504 (17.5)		
	Non-Hispanic Black	547 (10.6)	106 (12.0)	1 (0.5)	279 (24.1)	161 (5.6)		
	Non-Hispanic Unknown Race	58 (1.1)	11 (1.2)	0 (0.0)	6 (0.5)	41 (1.4)		
	Non-Hispanic White	3322 (64.5)	633 (71.4)	213 (98.2)	811 (70.0)	1665 (57.7)		
	Hispanic (All Races)	622 (12.1)	91 (10.3)	3 (1.4)	37 (3.2)	491 (17.0)		

¶ Non-Hispanic ethnicity was used as a reference
